# Strengthening the Diagnosis and Treatment of Malnutrition Through Increased Nurse Involvement: A Quality Improvement Project From Pediatric Wards in Mozambique

**DOI:** 10.9745/GHSP-D-23-00094

**Published:** 2023-12-22

**Authors:** Delfina Moçambique, Andreas Schindele, Osvaldo Loquiha, Sónia Martins, Monica Sequene, Amir Seni, Eugénia Macassa, Lara Samuel, Custódio Mondlane, Alex Vilanculo, Matias Epifanio, W. Chris Buck

**Affiliations:** a Hospital Central de Maputo, Maputo, Mozambique.; b University of Witten/Herdecke, Witten, Germany.; c Clinton Health Access Initiative, Maputo, Mozambique.; d Hospital Central da Beira, Beira, Mozambique.; e Pontifícia Universidade Católica Rio Grande do Sul, Porto Alegre, Brazil.; f David Geffen School of Medicine, University of California Los Angeles, Los Angeles, CA, USA.

## Abstract

**Background:**

Childhood acute malnutrition continues to be a serious health problem in many low-resource settings in Africa. On pediatric wards in Mozambique, missed opportunities for timely diagnosis and treatment of malnutrition may lead to poor health outcomes. To improve inpatient nutritional care, a quality improvement (QI) project was implemented that aimed to engage pediatric nurses in inpatient malnutrition diagnosis and treatment.

**Methods:**

In 2 Mozambican referral hospitals, for 6 months, the Plan-Do-Study-Act framework for QI was implemented to identify key drivers of the following measures: having complete anthropometric evaluation documented at admission, 3 or more weight measurements per hospitalization week, documentation of nutritional therapy for eligible patients, and documentation of referral for outpatient nutritional rehabilitation after discharge. Clinical data were abstracted from hospital charts and entered into an EpiInfo database, including a 3-month observation period after the project, and analyzed retrospectively.

**Results:**

A total of 2,208 children from wards other than malnutrition were included in the analysis. Complete anthropometric evaluation at admission improved from 24.4% 2 months before the QI project to 80.1% during and 75.2% in the 3 months after the project (*P*<.001). The percentage of patients with 3 or more weight measurements per hospitalization week rose from 22.3% to 82.8% during and 75.0% after the project (*P*<.001). Documentation of nutritional therapy increased from 58.8% before to 67.1% during and 70.6% after the project (*P*=.54), and documentation of referral for outpatient nutritional rehabilitation after discharge decreased from 55.9% to 54.9% during and increased to 70.6% after the project, (*P*<.001).

**Conclusion:**

Nurse engagement may lead to important advancements in the diagnosis and treatment of acute malnutrition in pediatric wards other than malnutrition in Mozambique. Task-sharing, particularly nurse engagement, in combination with QI methodology, may be considered for wards in similar settings with a high burden of malnutrition.

## Abstract (precis)

This study shows how increased nurse engagement combined with quality improvement methods may lead to important accomplishments in diagnosing and caring for malnourished children in pediatric wards in Mozambique.

## Teaser

**Key Findings:**

- Baseline performance in key malnutrition measures on pediatric wards other than malnutrition in 2 Mozambican referral hospitals was low, with only 24.4% of children adequately screened for malnutrition.
- A project for nurse engagement that used quality improvement methodology resulted in important advancements in nutritional screening and referral for outpatient nutritional rehabilitation after discharge.

**Key Implication:**

- Health policymakers and hospital medical and nursing leadership in resource-restrained settings may consider task-sharing, particularly nurse engagement, and quality improvement methods to strengthen care and treatment for malnourished children in pediatric wards.

## INTRODUCTION

Acute malnutrition in children continues to be a serious health problem in many low- and middle-income countries.^1^^,^^2^ It is defined by low weight for height, body mass index, or mid-upper arm circumference (MUAC), or by the presence of edema in the case of kwashiorkor.^3^^,^^4^ Major causes of acute malnutrition in children are poor diets/reduced calorie intake, infectious diseases including HIV, diarrhea, and malabsorption, and it is often associated with unfavorable socioeconomic conditions.^5^ When associated with other diseases, acute malnutrition considerably increases the risk of infant/child death.^6^^–^^8^ Worldwide, undernutrition, which comprises wasting, stunting, and underweight, is attributable to 45% of deaths in children aged younger than 5 years.^9^

Since 2020, the COVID-19 pandemic, conflict, and climate change have negatively affected food security worldwide. This threatens progress toward Sustainable Development Goal 2.2 to end all forms of malnutrition and Sustainable Development Goal 3.2.1 to reduce the mortality rate in children aged younger than 5 years.^10^ In 2022, 45 million children aged younger than 5 years were estimated to suffer from wasting (low weight for height) globally, with 13.7 million suffering from severe wasting.^1^ An estimated 12.2 million children with wasting live on the African continent, representing 27% of the global burden.^1^ In Mozambique, 200,800 children aged younger than 5 years were estimated to be wasted in 2020, corresponding to 3.9% of this age group.^1^

Whereas children with moderate or severe acute malnutrition without complications can be treated successfully at the community level, World Health Organization and Mozambican guidelines recommend that cases of severe malnutrition with complications be hospitalized for inpatient treatment.^3^^,^^4^^,^^11^ These children are typically admitted to malnutrition wards, where they receive focused nutritional rehabilitation as part of standardized treatment algorithms. However, in countries like Mozambique, there are often malnourished children in other pediatric inpatient wards that give less attention to the diagnosis and treatment of less severe forms of acute malnutrition, exposing these children to complications, including prolonged hospital stays, morbidity, and mortality.^8^^,^^12^^–^^15^ Mortality in young malnourished children has been shown to be elevated not only during hospital stay but also in the 6 months after discharge.^13^ Earlier intervention may prevent deterioration of the nutritional status of these children and the associated morbidity and mortality.

One challenge in the identification and treatment of malnutrition in hospitalized children in Mozambique relates to human resources and task distribution of ward teams. Staff shortages in the health sector affect the sub-Saharan Africa region disproportionally and are a limiting factor for quality of care in hospitals.^16^^,^^17^ Mozambique had 0.5 nurses working in the public health sector per 1,000 population in 2020, compared to 9.2 nurses per 1,000 in countries forming part of the Organization for Economic Co-operation and Development in 2021.^18^^,^^19^ Whereas 0.1 physician per 1,000 population worked in Mozambique in 2020, in Organization for Economic Co-operation and Development countries, the respective number was 3.7 per 1000 in 2021.^19^^,^^20^

One challenge in the identification and treatment of malnutrition in hospitalized children in Mozambique relates to human resources and task distribution of ward teams.

In 2018, an observation during a technical assistance visit to Hospital Central de Maputo found that the relatively small group of physicians and nutritionists responsible for nutritional screening were falling short of performance goals and that the larger group of inpatient nurses was an underutilized human resource that could likely contribute to improved inpatient nutritional care. In response to this feedback, a project making use of quality improvement (QI) methods was launched to engage pediatric nurses in the inpatient malnutrition diagnosis and treatment cascade on wards other than malnutrition in 2 of Mozambique's major central teaching hospitals. To demonstrate the potential impact of nurse engagement supported by QI methodology on nutritional diagnosis and treatment, a retrospective analysis of the project data was performed.

## METHODS

### Context

From May to November 2020, a project was implemented at the pediatric departments of Hospital Central da Beira (HCB) and Hospital Central de Maputo (HCM) to increase nurse engagement in acute child malnutrition in non-malnutrition wards, making use of QI methodology. These are the 2 principal referral hospitals for the Central and Southern regions of the country, respectively, but they also receive a large percentage of patients who present directly from home without referral. Both hospitals admit children aged 0–14 years.

On pediatric wards in Mozambique, 3 categories of nurses are usually employed: general nurses, pediatric nurses, and maternal and child health nurses. All 3 categories have further classifications as medium-level nurses with 2.5 years of training or superior-level nurses with 4.5 years of training, which includes more scientific content and a more specialized skillset.^21^^,^^22^

In 2020, there were 5,003 children admitted to the pediatric department of HCB, which was staffed by 37 doctors, 75 nurses, and 2 nutritionists, while the pediatric ward of HCM had 8,259 child admissions and staffing of 79 doctors, 138 nurses, and 3 nutritionists. As central referral hospitals, HCB and HCM have a larger concentration of physicians with a ratio of nurses to doctors of 2.0:1 and 1.9:1, respectively, while for the entire public health sector in Mozambique, the ratio is approximately 5:1.^19^

### Interventions

The project aimed to improve nutritional outcomes on pediatric wards other than malnutrition wards through increased engagement of hospital nursing staff.

At both hospitals, neonatology wards were not included in the project, as neonatal nutritional pathologies and respective care and treatment are distinct from acute malnutrition in older patients. Also, the pediatric intensive care units at both hospitals were excluded. Most other pediatric wards were included, namely general pediatric wards, infectious disease wards, and infant wards at both sites; at HCB, the respiratory disease ward was included, and at HCM, the pediatric surgery ward was included.

At the beginning of the project, nurses were given a 1-day training on nutritional screening/diagnosis, care, and treatment of acute child malnutrition, according to Mozambique Ministry of Health guidelines. Before the intervention, the nursing team was responsible for simply taking anthropometric measures at admission and reweighing patients during hospitalization, while doctors and nutritionists were responsible for using these measures to determine patients' nutritional status. As part of the QI project, the nurses were trained to use their anthropometric measurements to make acute nutritional status classifications.

To support the process of increasing nurse engagement in inpatient malnutrition diagnosis and treatment, QI methods were employed. QI comprises a broad range of efforts and methods to achieve positive outcomes and has been applied in multifaceted ways in the health sector.^23^^,^^24^ To support the nurses in the project, the Plan-Do-Study-Act (PDSA) framework seemed the most viable approach. It comprises 4 key iterative steps: “plan” relates to identification of problems and/or barriers and elaboration of a respective action plan, which is then implemented in the “do” step; outcomes are measured and interpreted in the “study” step to orient appropriate adjusting action in the “act” step.^25^

**FIGURE 1:**
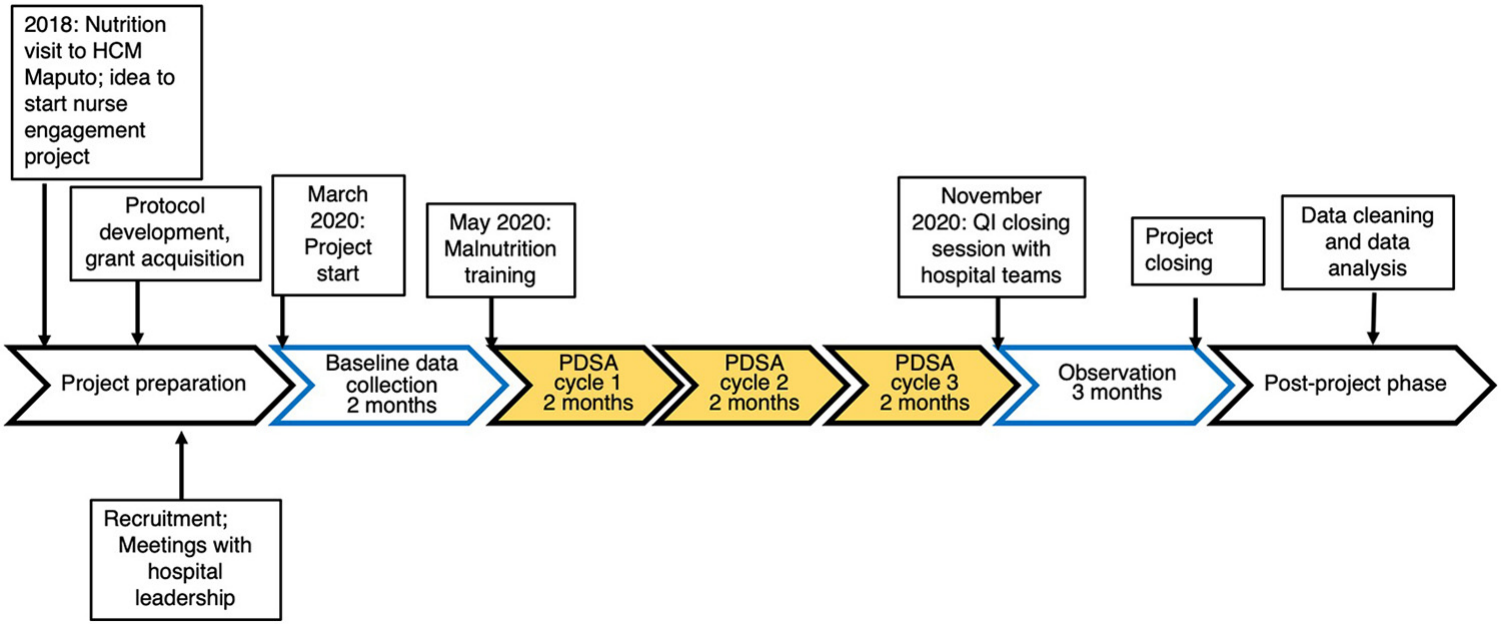
Timeline of the Quality Improvement Project to Improve Nutritional Outcomes on Pediatric Wards in 2 Referral Hospitals, Mozambique Abbreviations: HCM, Hospital Central de Maputo; PDSA, Plan-Do-Study-Act; QI, quality improvement.

After the initial malnutrition training, nurses participated in the first PDSA cycle session, grouped by their respective wards. At each site, nurses received a total of 3 PDSA cycle sessions, approximately 2 months apart. After the third PDSA cycle, the final results were presented to the hospital teams in a closing session. A timeline of the project is provided in Figure 1.

**FIGURE 2:**
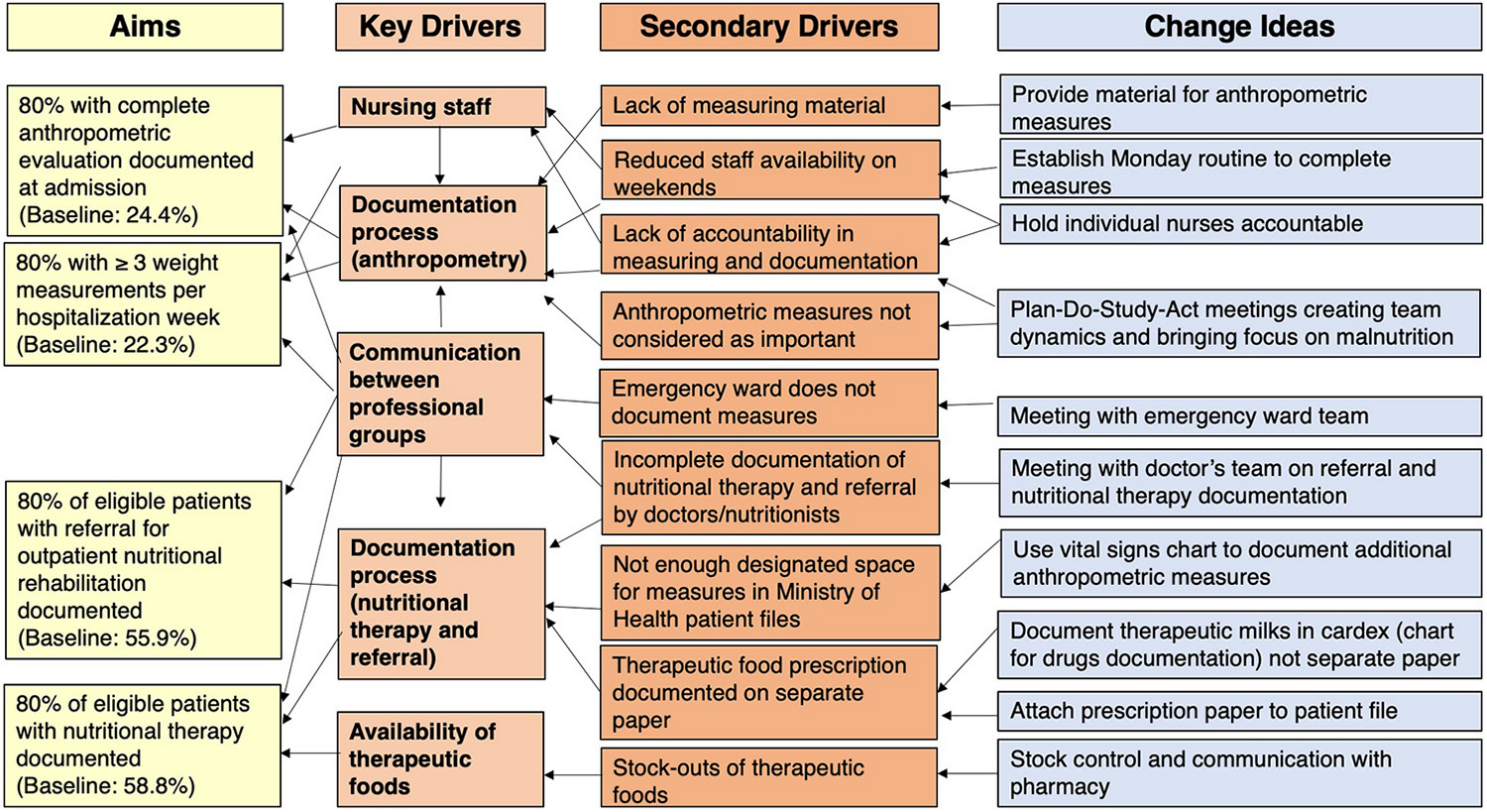
Key Drivers Influencing Process Measures and Outcome Measures of the Plan-Do-Study-Act Cycles in 2 Referral Hospitals, Mozambique

Each PDSA cycle was based on QI measures collected during a period of approximately 2 months before the corresponding PDSA cycle session. The sessions were moderated by a pediatric nurse and a pediatrician to accompany the ward nurse teams in the elaboration of action plans. Figure 2 shows a key drivers diagram depicting the process and findings of the PDSA framework as applied to the 4 selected QI measures. The team identified nursing staff, documentation process, and communication between different professional groups as key drivers influencing the 2 process measures of complete anthropometric evaluation documented at admission and proportion of patients with 3 or more weight measurements per hospitalization week. Communication between professional groups, documentation process, and availability of therapeutic foods were identified as key drivers impacting the 2 outcome measures of documentation of nutritional therapy for eligible patients and documentation of referral for outpatient nutritional rehabilitation after discharge.

At each site, the project's data assistants, a pediatric nurse at HCM, and a medical student at HCB, were available in case the nursing teams needed support for the implementation of the action plans. The head of pediatric nursing of both hospitals received a modest stipend for their role in project leadership, which required planning and coordination meetings with QI team leadership in addition to support of the ward teams at each hospital. All other nurses received a 1-off per-diem payment at the start of the project for their participation in the 1-day training course, which is normal practice in Mozambique for trainings outside of routine clinical work. They did not receive any additional compensation. No additional nurses were recruited for the project. The project had administrative support from the Clinton Health Access Initiative.

### Study of the Interventions

#### First PDSA Cycle (May–June 2020)

After the initial 1-day training in nutritional care and treatment, the study team presented baseline data from the 2 months preceding the project to the nursing teams, who, before this, had not been aware of the collection of data on nutritional screening and treatment. Nursing teams at both sites chose similar initial approaches, focusing on the improvement of communication flows and reinforcing responsibilities and accountability of tasks related to malnutrition. With assistance from the study team, nursing teams made sure that all necessary physical resources, including age-appropriate scales and nutritional evaluation tables, were available in all wards. Some wards established specific “measuring corners,” physical spaces for anthropometric measurement. Key activities were included in action plans for the wards, and regular meetings of nursing teams were established to monitor progress.

Nursing teams at both sites focused on improving communication flows and reinforcing responsibilities and accountability of tasks related to malnutrition.

To improve performance in nutritional screening, teams at both sites developed a key action point to hold the respective nurses accountable for complete anthropometric measures whenever they admitted a child on their ward. Ward nursing leadership also held meetings with nurses who worked in the urgent/emergency care units through which all children pass before admission to the ward to improve the quality, consistency, and documentation of anthropometric measurements at the time of admission. As the 4-page standard Mozambican inpatient paper chart does not have fields for routine documentation of complete anthropometric measures, nurses decided to use vital signs pages, which they routinely insert into inpatient charts for documentation of their anthropometric measurements and nutritional assessments.

Insufficient staffing during the weekend was identified as a reason for missing measures for children admitted during this time period, so nurses established a routine to review such patient files every Monday for completeness of anthropometric measures. As doctors also need access to patient files for their documentation responsibilities, nursing teams held meetings with the doctor teams to harmonize access to the patient files for the different professional groups so that nurses were able to document anthropometric measures.

#### Second PDSA Cycle (July–August 2020)

During the second cycle, accountability of individual nurses for anthropometric measurements and closer cooperation with the emergency ward were maintained as these were considered to have resulted in significant improvement in the completeness of documentation of anthropometric measures at admission and in the frequency of weight measurements on the wards. To achieve further improvements, nursing teams decided to further reinforce the Monday routine so that anthropometric measurements were performed for children admitted during the weekend. They also established practices to take measurements on patients who were bedridden and were not getting routinely reweighed.

Nursing teams identified the lack of appropriate documentation as the key challenge to improve the measure “documentation of nutritional therapy for eligible patients.” Medical doctors and/or nutritionists used to hand notes to patients for them to receive therapeutic milk, but often, this information was not recorded in the chart as required. So, nursing teams invited doctors and nutritionists to participate in the upcoming PDSA cycle meeting to discuss this topic. Another challenge was a temporary stock-out in therapeutic milk at HCB for 2 weeks. Nursing leadership contacted the pharmacy team to accelerate the administrative and logistical process to ensure the availability of therapeutic foods.

#### Third PDSA Cycle (September–October 2020)

Key action points of using the Monday routine to catch up with weekend admissions not yet screened for malnutrition and holding individual nurses accountable for anthropometric measurements of their patients brought improvements in the measures of anthropometric evaluation at admission and regular weight measurements, and these were maintained. Importantly, the 2 outcome measures related to documentation of inpatient nutritional therapy and referral for outpatient treatment after discharge were mainly under the responsibility of nutritionists and/or doctors. Communication between the nursing teams and the other professional groups did not show the expected improvement. So, during the third PDSA cycle, the QI team included nutritionists and medical doctors of the respective wards to participate in the PDSA cycle meetings to jointly identify solutions. At HCM, the patient treatment administration sheets on which drugs and therapeutic foods were registered were regularly separated from the patient file. When doctors did not actively search for these sheets when discharging a patient, the information went missing. So, it was agreed that the doctors always ensured that the patient treatment administration sheets were attached to the patient file at discharge.

Nursing leadership at both sites also complained that not all members of their teams showed the same motivation for the QI project and that some showed resistance to changing their daily routines. Nursing leadership addressed such issues by directly communicating with such team members.

### Measures

We selected the following 2 process measures for the PDSA cycles to represent key elements of the care and inpatient treatment cascade for acute child malnutrition.

- Complete anthropometric evaluation documented at admission, defined as weight, length/height, MUAC, and either weight for length/height or body mass index z-scores, documented within 24 hours after admission during workdays and within 72 hours for weekend admissions
- Proportion of patients with ≥3 weight measurements per hospitalization week

Improvements in nutritional screening at admission were identified as a priority for several reasons. First, timely nutritional screening is the prerequisite for any nutritional intervention. Furthermore, it needs a higher level of coordination of work tasks as it involves collecting and processing measurements of all children admitted and involves a set of tasks regularly performed by nurses in Mozambique. Due to evidence of poor performance during the pre-intervention period and because of its significance for ongoing nutritional evaluation during hospitalization, the measure related to repeat weight measurements during hospitalization was added.

To complement these 2 diagnostic-related process measures, the following 2 outcome measures were added to include essential steps in the care and treatment cascade for children once they are diagnosed with malnutrition.

- Documentation of nutritional therapy for eligible patients
- Documentation of referral for outpatient nutritional rehabilitation after discharge for eligible patients

### Data Collection

At both hospitals, data were collected retrospectively from patient files by a data assistant, in each case a health care provider with clinical working experience at the respective site. Data were entered into anonymized data collection sheets and an anonymized EpiInfo database. Data entry started 2 months before the beginning of the project and continued for 3 more months after the end of the project. All files of children hospitalized for at least 7 days were included. For patients with a hospital stay of less than 7 days, the data assistants tried to include all patient files. However, when the number of files of patients discharged from the wards was too high for the data assistant to enter all before handing them back to the hospital's archiving system, the data assistant randomized the files to enter (e.g., every second or third patient file only). After data collection had been finalized, data were systematically reviewed for consistency by a team of 2 pediatricians and a biostatistician. A total of 181 cases underwent corrections for either age or anthropometric measures, mostly by identifying data entry errors by cross-checking with the paper data collection forms. A further 21 cases were excluded for missing data or poor data quality, of a total of 2,229 cases.

### Analysis

Descriptive statistics for all numerical indicators were calculated and summarized by means of tables and graphs and comparisons made between the pre-intervention, intervention, and post-intervention phases. Comparative analyses were performed using differences in means or proportions via generalized estimating equations with Chi-squared tests to assess significance in results accounting for type of ward and site. Data were analyzed using R and Microsoft Excel 2016.

### Ethical Approval

The study was approved by the Scientific Directorates at HCM and HCB and the institutional bioethical review board of the Faculty of Medicine at Universidade Eduardo Mondlane, Maputo, Mozambique (registered under CIBS FM&HCM/045/2021). Caregiver informed consent was not required.

## RESULTS

A total of 2,208 children were included in the analysis:1,054 from HCM and 1,154 from HCB. A total of 426 children were included from the 3 months before implementation of the project, 1,254 during the project, and another 528 children during the 3 months after the end of the project. The median age was 36 months (95% confidence interval=11, 84 months), and 41.2% of patients were female. Age and sex did not differ significantly between the 3 phases of the QI project. The proportion of children with HIV was significantly higher (*P*<.001) during the intervention phase (9.6%) compared to pre-intervention and post-intervention phases, 6.6% and 5.9%, respectively. Most children were discharged from the infectious disease wards (31.0%), infant wards (24.2%), and general pediatric wards (23.1%) of the 2 sites (Table 1).

**TABLE 1.** Sociodemographic Patient Characteristics and Ward Distribution by Quality Improvement Project Phase, 2 Referral Hospitals, Mozambique

Indicator	Pre-Intervention Phase, No (%) n=426	Intervention Phase, No. (%) n=1,254	Post-Intervention Phase, No. (%) n=528	Total, No. (%) n=2,208
Median age (IQR), months	24 (10–72)	36 (13–86)	36 (11–84)	36 (11–84)
Age group, years				
<1	117 (27.5)	292 (23.3)	143 (27.1)	552 (25.0)
1–4	165 (38.7)	468 (37.3)	184 (34.8)	817 (37.0)
5–9	82 (19.2)	276 (22.0)	125 (23.7)	483 (21.9)
10–14	61 (14.3)	213 (17.0)	76 (14.4)	350 (15.9)
Not recorded	1 (0.2)	5 (0.4)	0 (0.0)	6 (0.3)
Sex				
Female	186 (43.7)	514 (41.0)	210 (39.8)	910 (41.2)
Male	240 (56.3)	740 (59.0)	318 (60.2)	1,298 (58.8)
HIV status				
Positive	31 (7.3)	123 (9.8)	31 (5.9)	185 (8.4)
Exposed	21 (4.9)	22 (1.8)	6 (1.1)	49 (2.2)
Negative	258 (60.6)	793 (63.2)	334 (63.3)	1,385 (62.7)
Not recorded	116 (77.2)	316 (25.2)	157 (29.7)	589 (26.7)
Ward distribution				
Infant ward	112 (26.3)	285 (22.7)	137 (25.9)	534 (24.2)
General pediatrics	114 (26.8)	295 (23.5)	100 (18.9)	509 (23.1)
Infectious diseases	115 (27.0)	408 (32.5)	162 (30.7)	685 (31.0)
Respiratory diseases	31 (7.3)	128 (10.2)	56 (10.6)	215 (9.7)
Pediatric oncology	12 (2.8)	65 (5.2)	42 (8.0)	119 (5.4)
Pediatric surgery	42 (9.9)	73 (5.8)	31 (5.9)	146 (6.6)

Abbreviation: IQR, interquartile range.

**FIGURE 3:**
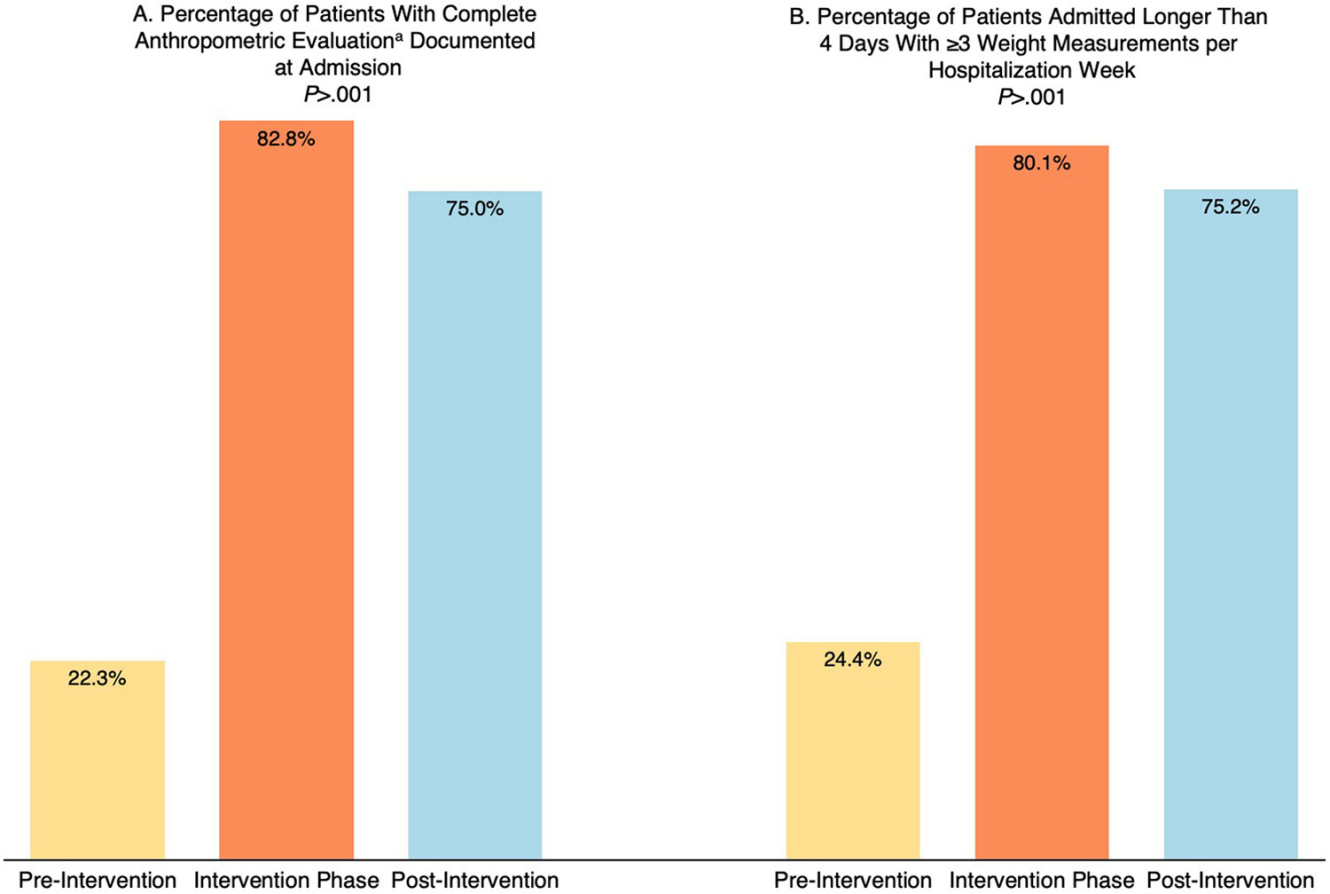
Evolution of Process Measures During Quality Improvement Project Phases in 2 Referral Hospitals, Mozambique ^a^ Defined as weight, length/height, mid-upper arm circumference, and either weight for length/height or body mass index z-scores, documented within 24 hours after admission during workdays, and within 72 hours for weekend admissions.

Table 2 shows the evolution of malnutrition-related indicators from pre-intervention to post-intervention. The priority process measure “complete anthropometric evaluation documented at admission” rose from 24.4% before the intervention to 80.1% during the intervention and maintained high performance at 75.2% during post-intervention follow-up (*P*<.001) (Figure 3). Critical submeasures to this key measure also showed significant improvement: documentation of height and MUAC at admission rose from 31.7% to 84.3% and from 27.7% to 70.0%, respectively (*P*<.001). Both measures showed sustained improvement throughout the post-intervention period, with 82.6% for height and 57.9% for MUAC documentation, respectively (both *P*<.001). “Documentation of admission weight” is the only measure that showed excellent performance before the intervention (98.8%), 99.2% during the project, and 99.8% during post-intervention follow-up. The only measure on anthropometric measurement performance without sustained improvement during the post-intervention period was “documentation of discharge weight,” rising from 54.5% pre-intervention to 90.5% during the project and dropping to 52.3% after the end of the project. However, when adjusted for systematically missing data during several weeks of the post-intervention period at HCB because the assistant at that site omitted this information from data collection, performance was maintained at 91.1% (*P*<.001).

**TABLE 2.** Evolution of Project Measures During Quality Improvement Project Phase, 2 Referral Hospitals, Mozambique

Measure	Pre-Intervention Phase, No. (%)	Intervention Phase, No. (%)	*P* Value^a^	Post-Intervention Phase, No. (%)	*P* Value^a^
Complete anthropometric evaluation^b^ documented at admission	104/426 (24.4)	1,005/1,254 (80.1)	<.001	397/528 (75.2)	<.001
Documentation of admission weight	421/426 (98.8)	1,244/1,254 (99.2)	.8962	526/528 (99.6)	.6468
Documentation of discharge weight	232/426 (54.5)	1,135/1,254 (90.5)	<.001	276/303 (91.1)^c^	<.001
Documentation of height (or length)	135/426 (31.7)	1,057/1,254 (84.3)	<.001	436/528 (82.6)	<.001
Documentation of MUAC	100/361 (27.7)	748/1,068 (70.0)	<.001	268/463 (57.9)	<.001
Average number of weight measurements per hospitalization week (SD)	2.72 (2.37)	6.4 (4.29)	<.001	5.78 (4.19)	<.001
Proportion of patients with ≥3 weight measurements per hospitalization week^d^	85/381 (22.3)	771/931 (82.8)	<.001	303/404 (75.0)	<.001
Documentation of nutritional therapy for eligible patients	20/34 (58.8)	55/82 (67.1)	.8684	24/34 (70.6)	.5392
Documentation of referral for outpatient nutritional rehabilitation after discharge for eligible patients	19/34 (55.9)	45/82 (54.9)	.1338	24/34 (70.6)	<.001

Abbreviations: MUAC, mid-upper arm circumference; SD, standard deviation.

a *P* values refer to comparison with the pre-intervention phase.

b Defined as weight, length/height, MUAC, and either weight for length/height or body mass index z-scores, documented within 24 hours after admission during workdays and within 72 hours for weekend admissions.

c Adjusted for missing values.

d Includes only patients that were admitted ≥4 days.

The second process measure, the number of weight measurements per patient per hospitalization week, improved significantly from 2.7 measurements per patient per week to 6.4 measurements per patient per week, also with sustained improvement after the intervention with 5.8 measurements (*P*<.001). The proportion of patients admitted for at least 4 days who had at least 3 weight measurements documented rose from 22.3% before the intervention to 82.8% during the intervention and 75.0% in the period after the intervention (*P*<.001) (Figure 3).

**FIGURE 4:**
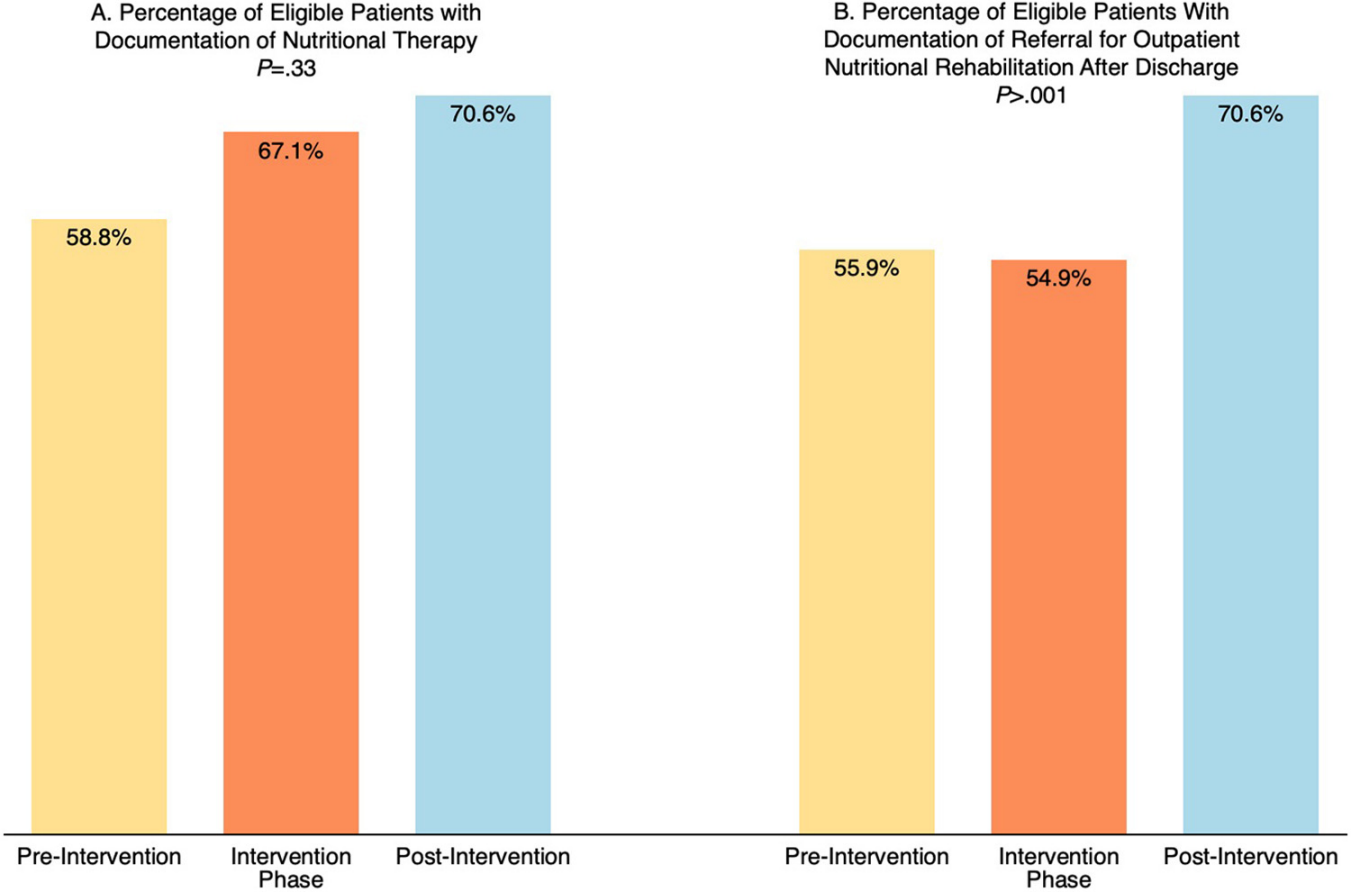
Evolution of Outcome Measures During Quality Improvement Project Phases in 2 Referral Hospitals, Mozambique

The first of the 2 outcome measures, “documentation of nutritional therapy for eligible patients,” rose from 58.8% before implementation to 67.1% during the project and improved further to 70.6% after the end of the project, without achieving statistical significance (Figure 4).

The second outcome measure, “documentation of referral for outpatient nutritional rehabilitation after discharge for eligible patients,” showed significant improvement only after the project, from 55.9% pre-intervention to 70.6% (*P*<.001) (Figure 4).

## DISCUSSION

This analysis demonstrated that a QI approach to increase nurse involvement can bring significant advancements in malnutrition care and treatment performance in pediatric wards in Mozambique. Most impressive improvements were noted in the priority process measure on completeness of documentation of anthropometric evaluation at admission, as well as in respective submeasures (e.g., weight measurements). This performance was maintained at high levels during the 3 months observation period after the project. Two factors may have additionally helped in bringing these impressive and lasting improvements via QI methodology: they are tasks within the scope of typical work for nurses, and they did not need cooperation between different professional groups.

Most impressive improvements were noted in the priority process measure on completeness of documentation of anthropometric evaluation at admission, as well as in respective submeasures.

Tasks that were principally physician or nutritionist responsibilities, namely the outcome measures on documentation of prescription of therapeutic foods and referral to outpatient nutritional rehabilitation, showed less impressive improvements and only improved later during the course of the project. This may be due to the late inclusion of medical doctors and nutritionists in a project mainly focused on the nursing teams. Early inclusion of all stakeholders and professional groups may have been more effective in achieving improvements in measures that depend on the respective professional groups.

Few studies have reported on nurse engagement to improve screening and care for malnourished children in low- and middle-income countries. A nurse mentorship intervention that sought to strengthen Integrated Management of Childhood Illness indicators in Rwandan primary health care centers proved successful in improving most of these indicators. However, rates of nutritional screening even worsened during the intervention, and it was perceived as a particularly challenging task for the nurses specifically trained in Integrated Management of Childhood Illness.^26^

There is scarce published evidence from sub-Saharan Africa on QI approaches in general, and more specifically, QI approaches in malnutrition, although the evidence is constantly increasing.^27^ Most published studies have come from South Africa and, in general, focus on entire hospitals or districts.^28^^–^^30^ Some results stress the importance of the working environment and team dynamics, but to our knowledge, no study has focused specifically on nurse engagement. A study from South Africa demonstrated how reductions in mortality from severe acute malnutrition in district hospitals can depend on favorable leadership and work culture.^29^ Another study from South Africa reported how the creation of an enabling local health system environment for maternal-child health positively influenced admissions for children aged younger than 5 years and in-hospital mortality for severe acute malnutrition in a 5-year district health system strengthening initiative.^30^

A recent child malnutrition QI project in a Malawian tertiary hospital reported high levels of coverage of nutritional assessment performed by dedicated lay staff, with 98.4% before and 97.1% during the QI intervention. However, the authors noted that the high performance may have been influenced by study data clerks who may have indicated to lay staff when nutritional assessment data were missing.^31^ In the Mozambican health system, task-shifting to lay staff has mainly happened in the area of HIV/AIDS due to more generous funding than in other areas.^32^^,^^33^ In the face of ongoing health budget restraints, task-sharing approaches such as the one in this project may be a more viable, flexible, and cost-effective option because it does not require hiring additional staff as task-shifting may require.^34^^,^^35^

In general, task-shifting approaches need to be weighed carefully with consideration of skill set, workload, and psychological aspects of the respective workforce.^36^ In this QI project, the new nursing tasks were mostly not new but part of the national nursing curriculum and were already being performed routinely by nurses in peripheral health centers, which may have facilitated the task-sharing approach used.

Measures were maintained at a high level of performance in the relatively short period of data monitoring after the end of the project, pointing to the potential of task-sharing/nurse engagement and QI approaches to induce sustainable improvements. Follow-up interviews with nursing leadership at both sites showed that in March 2022, 17 months after the end of the project, many of the adapted practices that led to the improvements noted during the QI project continued to be used, including routine reweighing of patients during hospitalization, practices to properly document anthropometric measures and nutritional therapy, and the ongoing use of the “measuring corners” that were established during the project.

### Limitations

This study has several limitations that need to be addressed. Regarding the sustainability of our results, a post-intervention observation period longer than 3 months would have provided more useful information. A study from South Africa was able to show that advances in severe acute malnutrition mortality achieved via a health system strengthening approach in 2 district hospitals were only slightly reversed in a 37-month observation period after the project.^28^ Some aspects point to the potential for sustainability of the project. A short-term monthly stipend was indeed paid to the 2 departmental nurse leaders at each hospital due to the significant additional work the QI project created for them, but after discontinuing the stipend after the project phase, performance was maintained for the 3 months. Ward nurses did not receive any additional compensation other than the routine per diem given for the single day of initial training. Clinical partners who are not routine members of the ward teams helped lead the project during the 6-month QI phase, but they did not contribute to any of the clinical activities, and no new hospital staff were hired.

A further limitation is that improvements in care and treatment for children with acute malnutrition should ideally result in reduction of inpatient and post-discharge mortality, but we were unable to assess mortality with our data set. It was also not possible to take into consideration the impact of any other programs or activities at the level of the Ministry of Health or either hospital during the time of the study that may have positively or adversely impacted pediatric inpatient nutritional care. Finally, the data used for analysis were collected as part of a QI project and not as part of a research protocol and, as such, were susceptible to quality problems. However, a thorough data review was performed with data cleaning when needed and exclusion of patients with key missing variables or inconsistent information.

## CONCLUSION

Nurse engagement supported by QI methodology proved to be an effective strategy to achieve important accomplishments in key elements of the malnutrition diagnosis and treatment cascade in pediatric wards in Mozambique. QI approaches may be further integrated into national programs in resource-constrained settings to improve early detection of hospitalized children who are malnourished and enable prompt treatment to prevent further degradation of nutritional status and associated complications during hospitalization.

## Notes

**Peer Reviewed**

**First published online:** XXX.
